# Mixed Use of Bio-Oil in Oil Power Plants: Should It Be Considered When Developing NH_3_ Emission Factors?

**DOI:** 10.3390/ijerph18084235

**Published:** 2021-04-16

**Authors:** Seongmin Kang, Jiyun Woo, Eui-Chan Jeon

**Affiliations:** 1 Climate Change & Environment Research Center, Sejong University, Seoul 05006, Korea; smkang9804@gmail.com; 2 Department of Climate and Environment, Sejong University, Seoul 05006, Korea; woojune92@gmail.com

**Keywords:** PM_2.5_ secondary sources, oil power plant, bio-oil mix, missing sources, ammonia emission factor

## Abstract

In order to cope with recent climate change, Korea is reducing the use of heavy oil in petroleum-fired power plants and mixing bio-oils. Accordingly, this must be taken into account when calculating the emissions of air pollutants. However, in the case of Korea, when calculating NH_3_ emissions, the United States Environmental Protection Agency (EPA) emission factor is applied as it is to calculate emissions, and for petroleum power plants, the heavy oil emission factor proposed by EPA is used as it is to calculate emissions. In petroleum power plants, bio-oil is not mixed in a certain amount and used at a different ratio depending on the situation of the power plant. Therefore, in this study, the NH_3_ emission factor according to the mixing ratio of bio-heavy oil is calculated and the mixing ratio is calculated. As a result of the analysis, the emission factor according to bio-oil and the mixed ratio was found to be in the range of 0.010~0.033 kg NH_3_/kL, and it was lower than the heavy oil emission factor 0.096 kg NH_3_/kL of EPA currently used in Korea. This is because the amount of NH_3_ through the slip is also small since the use of NH_3_ for reduction is also low because the NOx emission from the use of bio-oil is low. Considering all of these points, we have statistically analyzed whether emission factors should be developed and applied. As a result of the confirmation, the difference according to the mixed consumption rate was not large.

## 1. Introduction

Recently, as the use of fossil fuels has increased, problems associated with climate change have become serious. Air pollution from PM_2.5_ (particulate matter less than 2.5 µm^3^), in particular, has been increasing steadily, endangering human health [1,2,3]. Sources of direct emissions of such PM_2.5_ have been relatively well managed as a result of the efforts of many policies. However, in the case of substances caused by secondary generation, we are only recently paying attention and making efforts to reduce. [4] In South Korea, emissions of NOx and SO_X_—well-known PM_2.5_ secondary sources—have been managed properly based on policies; however, there have not been many studies on ammonia(NH_3_) [5,6,7,8]. For example, Korea uses NH_3_ emission factors from other countries to calculate emissions and has many missing sources for NH_3_.Therefore, the NH_3_ inventory needs improvement, including in terms of NH_3_ emissions factor development and the estimation of emissions levels reflecting characteristics of South Korea.

Power plants are among the sources of NH_3_ emissions in South Korea. They use selective catalytic reduction (SCR) and selective non-catalytic reduction (SNCR) as preventive measures to reduce emissions of air pollutants such as NOx. SCR and SNCR are methods of reducing NOx levels using NH_3_; however, if NH_3_ is used excessively for NOx reduction, slip may occur [9,10,11,12,13]. Therefore, in the case of power generation facilities, the amount of NH_3_ discharged to the final outlet due to slip is calculated.

NH_3_ emissions from oil-fired power plants are computed with the NH_3_ emission factor for heavy oil used by the United States Environmental Protection Agency (US EPA). However, the use of heavy oil in oil-fired power plants has recently been on a decline in South Korea. This is because many power plants are generating power by mixing bio-oil in their fuel or using 100% bio-oil as renewable energy in response to climate change. Bio-oil is a biofuel made by reacting animal and vegetable fats with methanol or ethanol and has similar characteristics to heavy oil, so it can be used by mixing with heavy oil in existing heavy oil power plants or replacing heavy oil [14]. Bio-oil is used for fuel conversion in terms of climate change because CO_2_ emitted from use due to carbon neutrality is not calculated as total emissions [15].

Therefore, it is necessary to consider an NH_3_ emission factor that takes the use of bio-oil into account [16,17,18]. However, in the case of bio-oil mixed consumption, it is different for each power plant, and even at the same emission facility, the mixed consumption rate is applied differently according to the situation of the power plant.

In this study, the NH_3_ emission coefficient was calculated based on the mixing rate of bio-oil and the 100% use of bio-oil. In addition, it was investigated through statistical analysis whether the mixed consumption ratio of bio-oil, which is applied in various ways, should be considered when developing NH_3_.

## 2. Methods

### 2.1. Selection of Objective Facilities

In this study, three facilities are used as case studies to determine whether the ratio of bio-oil to heavy oil should be considered when developing an emission factor for NH_3_ emitted from oil-fired power plants. In the case of oil power plants that use bio-oil, the same ratio is not applied, so the mixing ratio applied at the time of field measurement was divided into sections. NH_3_ concentration analysis was conducted on 38 samples. Table 1 shows how the number of samples collected for each bio-oil and heavy oil mixing ratio was classified. The greatest number of samples collected represented 100% bio-oil usage because many power plants in South Korea use bio-oil blends or bio-oil alone compared rather than only heavy oil.

### 2.2. NH_3_ Analysis at Mixed Use of Bio-Oil in Oil Power Plants

**Figure 1:**
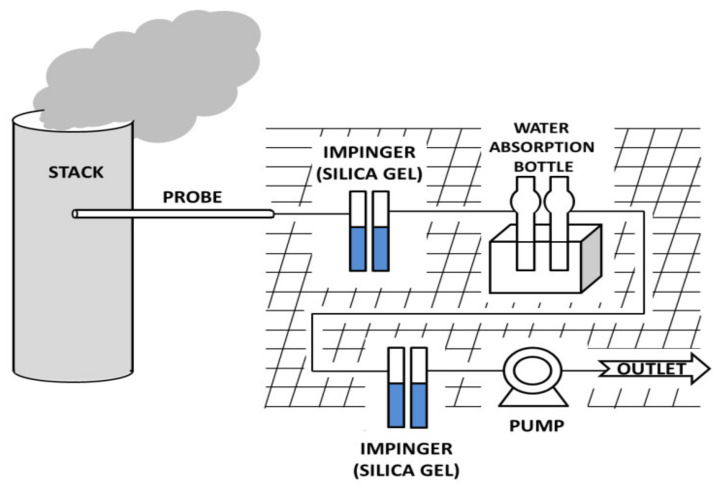
Schematic of the field setup for ammonia sampling at oil power plant.

The samples in this study were collected in accordance with the standard testing method used in South Korea to measure the concentration of NH_3_ emissions from oil-fired power plants. The indophenol method was used to collect ammonia samples for this analysis [19,20,21]. To collect the ammonia samples, an ammonia-absorbing solution was created using a total of 50 mL boric acid solution and poured into two flasks (50 mL each); a mini-pump (Shibata MP-ΣNII, Japan) was then used to introduce 80 L of exhaust gas into these flasks at a rate of 4 L/min for about 20 min. Exhaust gas from power plants contains a large quantity of water; because NH_3_ is easily absorbed in water, an absorption bottle containing silica gel was installed to minimize its effect [22,23]. Figure 1 shows how NH_3_ samples were collected. Phenol-sodium nitroprusside solution and sodium hypochlorite solution were added to the solution onto which NH_3_ was absorbed; the amount of ammonia present was calculated by measuring the absorbance of the indophenols that were produced via reactions with ammonium ions [24]. A spectrophotometer (Shimadzu 17A, Japan) was used to measure the absorbance at the 640 nm wavelength.

### 2.3. Development of NH_3_ Emission Factor

The equations used in two related studies on the development of the NH_3_ emissions factors were used to calculate the emissions factor for NH_3_; the method used in Equation (1) was also utilized [25,26]. NH_3_ concentration, flow rate, and oil usage are required to calculate the NH_3_ emissions factor for an oil-fired power plant. Flow rate data were received from the target power plant. The amount of oil used on the date of measurement was also used by receiving data from the target power plant.

(1) $$EF_{NH_{3}}=\left[C_{NH_{3}}\times \frac{M_{w}}{V_{m}}\times Q_{day}\times 10^{-6}\right]/FC_{day}$$

where EF is emission factor (kg NH_3_/kL); $C_{{\mathrm{NH}}_{3}}$ is *NH_3_* concentration in exhaust gas (ppm); $M_{w}$ is molecular weight of *NH_3_* (constant) = 17.031 (g/mol); $V_{m}$ is one mole ideal gas volume in standardized condition (constant) = 22.4 (10^−3^ m^3^/mol); $Q_{day}$ is daily accumulated flow rate (Sm^3^/day) (based on dry combustion gas); and $FC_{day}$ is daily oil consumption (ton/kL).

### 2.4. Statistical Analysis Method

**Figure 2:**
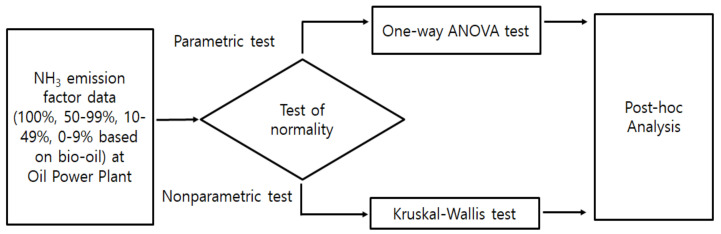
Schematic of statistics analysis.

This study compared the mean distribution of NH_3_ emission factors against different oil mixing ratios to determine whether it was affected by the mixing ratio of bio- and heavy oil. The statistical analysis was performed using the software SPSS 21(IBM, Armonk, NY, USA), and Figure 2 shows the statistical procedure used to analyze the difference in the NH_3_ emissions among different mixing ratios.

## 3. Result and Discussion

### 3.1. NH_3_ Emission Factor at Oil Power Plant

In this study, the NH_3_ emission factor for each section of the mixed consumption ratio was calculated to determine the factors influencing the NH_3_ emission factor according to the bio-oil mixing ratio of oil power plants, and it is shown in Table 2.

The results of these calculations showed that the NH_3_ emissions factor was 0.010 kg NH_3_/kL with a standard deviation of 0.018 kg NH_3_/kL when 100% bio-oil was used and 0.011 kg NH_3_/kL with a standard deviation of 0.010 kg NH_3_/kL when 50–99% bio-oil was used. Furthermore, when the bio-oil was mixed at a ratio of 10–49%, the NH_3_ emissions factor was 0.034 kg NH_3_/kL with a standard deviation of 0.025 kg NH_3_/kL and when the mixing ratio was 0–9%, the NH_3_ emissions factor was 0.033 kg NH_3_/kL with a standard deviation of 0.016 kg NH_3_/kL. As a result of the estimate by NH_3_ emission factors, the lower the bio-oil mixing ratio (when the heavy oil ratio was high), the higher the NH_3_ emission factor. NH_3_ emission at a power plant is related to reducing NOx [27,28,29]. Some studies show that less NOx and SOx are generated when bio-oil is used compared to when ordinary heavy oil is used in oil-fired power plants [30,31]. Therefore, when bio-oil is used as fuel, because NOx emission is low, only a small amount of NH_3_ is used for NOx reduction; this leads to a relatively low slip and, consequently, a low NH_3_ emissions factor. The deviation was large overall. Each deviation is determined to be based on bio-oil content rather than facility-based, the conditions at combustion of the facilities are not the same, and some items are divided into sections and are averaged, so the relevant deviation is considered large.

The NH_3_ emissions factor developed using the bio-oil mixing ratio was compared to that developed using heavy oil because it was difficult to find results from similar studies to use for comparison purposes. The results of the comparison show that the NH_3_ emissions factor for the oil-fired power plant calculated in this study was lower than that used by the US EPA, which is 0.096 kg NH_3_/kL. This difference is similar to the results of the previous study, because the NOx emission by the use of bio-oil is lower than that of heavy oil, so the use of NH_3_ for NOx reduction is less, and the emission by slip is lowered. Therefore, it is judged that the NH_3_ emission factor due to bio-oil mixing is lower than that of the heavy oil NH_3_ emission factor.

Korea is currently using the US EPA’s heavy oil NH_3_ emission factor. However, the actual measurement results show that there are differences and do not reflect the situation in Korea. Therefore, this study determined that an NH_3_ emissions factor reflecting the conditions in South Korea should be developed.

### 3.2. Kruskal–Wallis Test of Bio-Oil Mixed Rate in Oil Power Plant

Before a statistical analysis can be conducted, a first analysis should be performed to determine whether parametric or non-parametric methods of the former should be used [32,33]. However, if the number of data points is less than 30, any statistical analyses conducted are assumed to be non-parametric [34,35]. Therefore, in terms of a method of comparing the mean distribution of data, a non-parametric test was assumed to be necessary because the number of data points based on the mixing ratio of bio-oil to heavy oil was less than 30. Thus, the Kruskal–Wallis test—a non-parametric test—was used in this study. Its results are shown in Table 3.

To investigate the effect of the bio-oil mixing ratio on the NH_3_ emissions factor, the mean distribution of the latter was compared using the Kruskal–Wallis test.

In the results of the Kruskal–Wallis test, the significance of the probability (the *p*-value) was greater than 0.05; this indicated that the null hypothesis, “there is no difference in the NH_3_ emission factor between different mixing ratios,” was maintained. This means that the mixing ratio of bio-oil to heavy oil does not have much impact on the NH_3_ emissions factor at the oil-fired power plants where bio-oil is used in the fuel.

## 4. Conclusions

Ammonia must be managed to reduce PM_2.5_ emissions. However, in the case of Korea, emission factors of the US EPA are applied when calculating ammonia emissions, and there are also many emission factors that are currently omitted. In the case of oil-fired power plants, emissions are estimated by applying the US EPA NH_3_ emissions factor for heavy oil, but estimates of NH_3_ emissions for South Korea should consider that many facilities in the country currently use a bio-oil/heavy oil mixture or 100% bio-oil to run power plants in response to climate change. Furthermore, because the bio-oil mixing ratio is different depending on the situation at individual power plants, this study also used statistical analysis to determine whether that fact should be considered when developing NH_3_ emissions factors for oil-fired power plants.

This study classified mixing ratios based on the proportion of bio-oil in the fuel blend and developed NH_3_ emission factors for 3 power plants using a total of 38 samples. The results of the analysis demonstrated that the NH_3_ emission factor increases with the increase in the proportion of heavy oil in the fuel blend increases. It was found that because bio-oil combustion emits less NOx than heavy oil combustion, the less NH_3_ usage to reduce NOx levels than heavy oil does. This leads to a relatively low NH_3_ slip; therefore, the emissions factor is low. Furthermore, the results of this study and the comparison with the US EPA heavy oil NH_3_ emission factor currently applied in Korea showed that the NH_3_ emission factor of this study calculated by actual measurement was lower than that currently applied in Korea. This implies that South Korea should develop and apply NH_3_ emissions factors that reflect the characteristics relevant to its situation.

In addition, a statistical test was used in the analysis of whether the mixing ratio should be considered as part of the process to determine the NH_3_ emissions factor. Non-parametric analysis was conducted because there were fewer than 30 samples in each section of the range of mixing ratios; furthermore, because there were 4 mixing ratios—a total that was greater than 3—the analysis method used was the Kruskal–Wallis test (a non-parametric group comparison method). The results of this analysis suggested that the null hypothesis, “there is no difference in NH_3_ emission factor between mixing ratios of bio-oil,” should be maintained. This indicates that the mixing ratio does not have a significant impact on the development of emissions factors.

The contents and meanings that can be confirmed through this study are as follows:

1. Regarding NH_3_ emissions due to bio-oil mixed combustion, which does not reflect current NH_3_ emissions, NH_3_ emissions were checked and an emission factor was calculated.
2. In the case of NH_3_ emissions from oil power plants, Korea calculates emissions only for heavy oil, so this study presents matters related to bio-oil use and divides the mixed ratio into sections to represent NH_3_ emission factors.
3. Currently, in the case of Korea, only the emission factor of the US EPA’s heavy oil NH_3_ is applied and the emission is calculated. Therefore, it is suggested that there is a difference by calculating and comparing the emission factor according to the mixed consumption rate. In addition, through this, the necessity of developing emission factors reflecting the characteristics of Korea was mentioned.
4. It was confirmed whether it was necessary to consider all the mixing ratios by statistically analyzing the difference in the NH_3_ emission factor according to the bio-oil mixing ratio. As a result of the confirmation, the difference according to the mixed consumption rate was not large, so the necessity of developing the emission factor according to the representative bio-oil mixed consumption was suggested.

In the future, the reliability of the NH_3_ inventory will be improved if NH_3_ emissions factors are developed by investigating the uncertainties related to or characteristics of the emissions factors in addition to the number of samples taken and power plants in operation. That would then contribute to the establishment of policies to reduce pollution from PM_2.5_.

## Figures and Tables

**Table 1:** Sampling status of objective facilities.

Objective Facilities	Mixed Rate	Sampling
Oil Power Plant	100%	19
	50–99%	7
	10–49%	8
	0–9%	4
Total		38

**Table 2:** NH_3_ emission factor of bio-oil mixed rate in oil power plant.

Objective Facilities	Mixed Rate Based Bio-Oil	This Study (kg NH_3_/kL)	SD (Standard Deviation) (kg NH_3_/kL)	Sampling	EPA (1994) Based Heavy Oil (kg NH_3_/kL)
Oil Power Plant	100%	0.010	0.018	19	0.096
	50–99%	0.011	0.010	7	
	10–49%	0.034	0.025	8	
	0–9%	0.033	0.016	4	

Note: EPA: Environmental Protection Agency.

**Table 3:** The result of Kruskal–Wallis test by NH_3_ emission factor for bio-oil mixed rate.

Hypothesis Test	Null Hypothesis	Test	Sig.	Decision
NH_3_ emission factor for bio-oil mixed rate	The distribution of NH_3_ emission factor is the same across categories of bio-oil mixed rate	Independent Samples Kruskal–Wallis Test	0.148	Retain the null hypothesis
